# Breaking new ground in antimicrobial stewardship in companion animal veterinary practice

**DOI:** 10.1038/s41467-021-22542-0

**Published:** 2021-04-27

**Authors:** David Brodbelt

**Affiliations:** Veterinary Epidemiology, Economics and Public Health Group, PPS Department, Royal Veterinary College, Hawkshead Lane, Hatfield, AL9 7TA Herts UK

**Keywords:** Antibiotics, Antimicrobial therapy, Decision making, Human behaviour

## Abstract

Singleton and colleagues publish in *Nature Communications* an intervention study to reduce antimicrobial usage in companion animal practice. They identify significant reductions in antimicrobial usage with their more active intervention group over approximately a 6-month period. The study offers an exciting way forward to explore further the trial interventions and assess alternative methods to improve antimicrobial stewardship in veterinary practice.

## Antimicrobial usage in companion animals

Antimicrobial resistance (AMR) is an increasing concern in veterinary medicine, with much attention given to the role of antimicrobial (AM) usage in production animals in the development of AMR in both animals and people. More recently, there has been growing interest in AM usage in companion animal veterinary practice, an environment in which AMs are also regularly prescribed and dispensed. Recent papers have reported on AM usage in companion animal practice and highlighted as of particular concern the use of certain AMs that are considered in human medicine as second- or third-line therapeutics^1–6^. These therapeutics are termed critically important AMs (CIAs) by the World Health Organisation (WHO) based on evidence of developing AMR and limited alternative choices to treat serious bacterial infections^7^. Of special note, the highest priority CIAs including third-generation cephalosporins, potentiated penicillins and fluoroquinolones, which are reserved in medicine for treatment of life-threatening infectious conditions, have been reported to be frequently prescribed in veterinary practice^1–5,8^. Though many of these studies did not assess the appropriateness of the AM usage reported, they have highlighted the opportunity to consider reduction of AM usage generally and of strategically important CIAs to help preserve AM effectiveness.

## Antimicrobial stewardship in veterinary practice

It is therefore important that veterinarians use the right type of AM, at the correct dose, at the right time: a concept known as AM stewardship. AM stewardship is an important plank in the fight against AMR, and central to this stewardship is the need for clear guidelines on AM usage. This need for guidelines has been recognised by a number of veterinary organisations. In companion animal veterinary medicine, a major UK-focused initiative launched by the British Small Animal Veterinary Association (BSAVA) developed guidelines within the PROTECT ME initiative^9^. In Europe, the Federation of European Companion Animal Veterinary Associations (FECAVA) has produced a series of four recommendations in poster format dealing with practice hygiene, prudent AM usage, therapeutic approaches to common small animal infections, and advice to pet owners^10^. Work from a veterinary teaching hospital suggested that implementation of such guidelines for AM usage can improve prescribing behaviour^11^. However, recent qualitative evidence indicated that veterinary practitioners often have limited awareness of the guidelines^12,13^.

Guidelines and other measures are expected to reduce unnecessary AM usage and enhance therapeutic success of treated animals, thus reducing the burden of infectious diseases and the occurrence and spread of AMR. However, currently their impact on AM usage behaviours is largely unknown^14^. Recent work in the Netherlands reported reduced AM usage after recruitment of practices to an AM stewardship programme^15^. This study involved a relatively small set of highly motivated practices, and included some financial remuneration for participants, making it difficult to assess how scalable such approaches might be to veterinary practice in general. With the growing awareness of the need for improved AM usage, it is increasingly important to develop further the evidence base for optimal strategies to promote stewardship of AMs in veterinary practice.

## An intervention to reduce antimicrobial usage in companion animal practice

In their *Nature Communications* article^16^, Singleton and colleagues make a major inroad to begin to address the challenge to improve AM stewardship in companion animal veterinary practice. Using a randomised controlled trial approach, the authors evaluated short-term changes in AM usage within a control group and two intervention strategies. They undertook these interventions in groups of approximately 20 practices per study group within a corporate group in the UK, and monitored the practices’ AM usage over time for approximately 6 months after onset of interventions. The study identified associations between intervention type and subsequent AM usage levels, with certain interventions associated with a reduction in usage in participating practice. I encourage the interested reader to go to the full paper^16^ to see their specific findings.

The study^16^ offers some valuable insights into how to go forward to continue to improve AM stewardship in veterinary practice, and the authors should be congratulated for their work. They do acknowledge that the assessments over time were short term, and future work would need to evaluate whether the study and other interventions improve AM prescribing behaviour longer term in a sustainable manner.

As for much of the AM usage work in veterinary medicine, the study^16^ was not focused on evaluating appropriateness of usage but more on methods to reduce usage in general and of the highest priority CIAs in particular. These objectives are important but are not the whole picture, and the balance between the need to continue to work to reduce AM usage in veterinary practice where possible must be considered against the preservation of veterinarians’ ability to treat appropriately infectious diseases and relieve suffering. Singleton and colleagues^16^ offer an interesting insight into the route forward and lay the ground for further work to tease out this challenging and important field.
